# Disentangling optically activated delayed fluorescence and upconversion fluorescence in DNA stabilized silver nanoclusters†
†Electronic supplementary information (ESI) available. See DOI: 10.1039/c9sc00865a


**DOI:** 10.1039/c9sc00865a

**Published:** 2019-04-24

**Authors:** Stefan Krause, Cecilia Cerretani, Tom Vosch

**Affiliations:** a Nanoscience Center , Department of Chemistry , University of Copenhagen , Universitetsparken 5 , Copenhagen 2100 , Denmark . Email: stefan.krause@chem.ku.dk ; Email: tom@chem.ku.dk

## Abstract

Optically activated delayed fluorescence (OADF) is a powerful tool for generating background-free, anti-Stokes fluorescence microscopy modalities. Recent findings, using DNA-stabilized silver nanoclusters (DNA-AgNCs), indicate that OADF is usually accompanied by a dark state-mediated consecutive photon absorption process leading to upconversion fluorescence (UCF). In this study, we disentangle the OADF and UCF process by means of wavelength-dependent NIR excitation spectroscopy. We demonstrate that, by appropriate choice of secondary NIR excitation wavelength, the dark state population can be preferentially depleted through OADF, minimizing the UCF contribution. These findings show that dark state depletion by OADF might enable background-free STED-like nanoscopy.

## Abstract 2

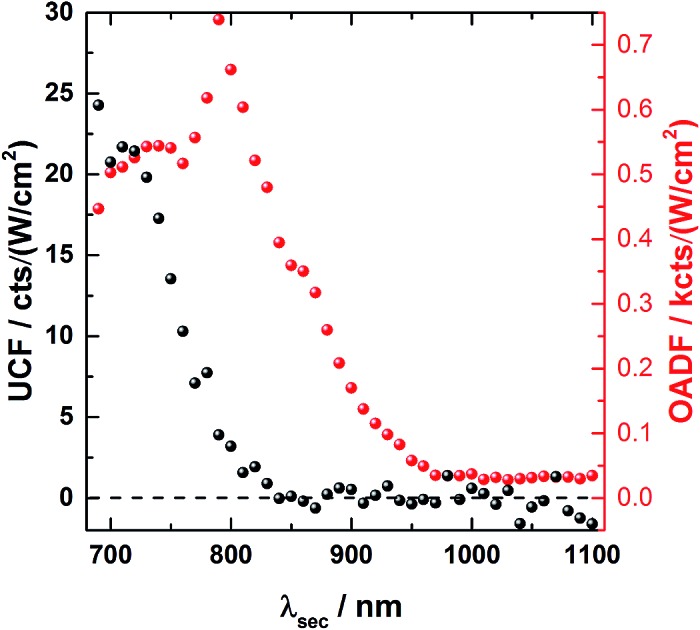
Optically activated delayed fluorescence (OADF) is a powerful tool for generating background-free, anti-Stokes fluorescence microscopy modalities.

## Introduction

Autofluorescence of tissues and cells is a common problem in fluorescence microscopy, as it can compete with the fluorescence signal of interest (*e.g.* in single molecule studies) or complicate signal quantification. Methods developed to mitigate some of these problems are two-photon microscopy,^1^ lanthanide-based upconversion fluorescence,^2^,^3^ time-gated imaging,^4^,^5^ and optical modulation techniques,^6^–^8^ among others. Recently, Fleischer *et al.* introduced optically activated delayed fluorescence (OADF), which can successfully remove unwanted background fluorescence.^9^,^10^ The OADF approach has been demonstrated so far only using DNA-stabilized silver nanoclusters (DNA-AgNCs) by taking advantage of their particular photophysical properties.^9^–^11^ DNA-AgNCs are a new class of emitters, first introduced by Petty *et al.* in 2004.^12^,^13^ After excitation with visible light, DNA-AgNCs can enter a microsecond long-lived dark state.^7^,^14^–^17^ Subsequent irradiation with NIR light can transfer the DNA-AgNCs back into the emissive state where fluorescence will appear on the nanosecond timescale.^9^–^11^ In combination with time-gating, the primary fluorescence (PF), occurring after the visible excitation pulse, can be separated from the secondary NIR pulse-induced OADF emission. Since OADF appears on the anti-Stokes side with respect to the NIR pulse, Krause *et al.* demonstrated that this technique can be readily used for background-free imaging.^10^ However, high intensities of the NIR secondary read-out beam can cause upconversion fluorescence (UCF). In this paper, we use secondary NIR excitation spectroscopy to disentangle the OADF from the UCF contribution. This wavelength-dependent information allows to select preferentially OADF and minimize UCF. Such ability is of significant importance since specific depletion of the dark state by OADF can facilitate future applications of OADF for STED-like optical nanoscopy.^18^

## Results and discussion

### Unraveling OADF from UCF

**Fig. 1:**
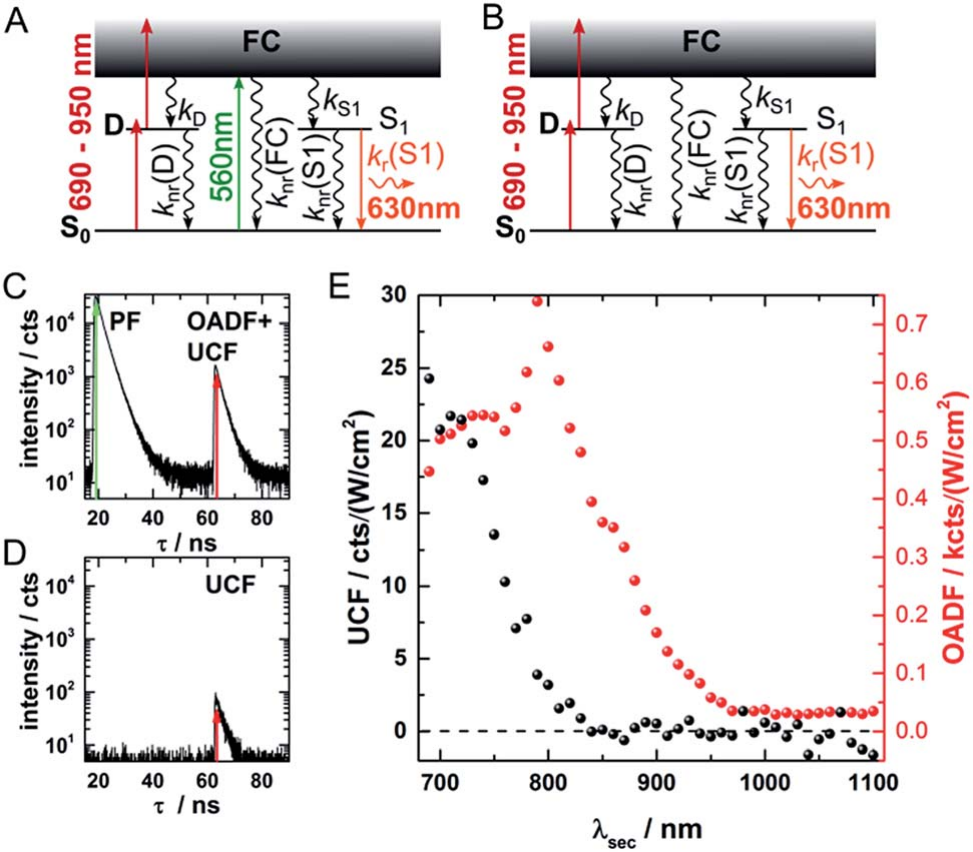
(A) Energy diagram of DNA-AgNCs embedded in PVA and the OADF + UCF excitation scheme. Vertical, colored arrows indicate the absorption of a visible (green arrow) or NIR (red arrows) photon and the emission of a visible photon (orange arrow). (B) Energy diagram of DNA-AgNCs embedded in PVA and the UCF only excitation scheme. Vertical, colored arrows indicate the absorption of two subsequent NIR photons (red arrows) and the emission of a visible photon (orange arrow). (C) Primary fluorescence (PF) decay curve starting at 20 ns upon 560 nm excitation and OADF + UCF decay curve starting at 63 ns after secondary 690 nm excitation. (D) UCF decay curve at 63 ns after subsequent absorption of 690 nm photons. (E) UCF (black) and OADF (red) intensity as a function of secondary excitation wavelength *λ*_sec_. The intensities were corrected for the varying secondary excitation intensity. Acquisition time for each data point was 100 seconds.

Fig. 1 provides an overview of the excitation schemes used to disentangle OADF from UCF. 560 nm pulsed excitation light was selected from a continuum laser (FWHM about 50 ps, 11.13 MHz repetition rate) and focused onto the sample to excite the DNA-AgNCs to a short-lived Franck–Condon (FC) state.^13^,^15^,^19^,^20^ From the FC state, a small fraction of DNA-AgNCs in the excitation volume can migrate into a microsecond long-lived dark state D. The majority of DNA-AgNCs will populate the emissive state (denoted S_1_ in analogy to organic dye photo physics) from which they relax back to the ground state (S_0_) on a nanosecond timescale, predominantly by fluorescence. This primary fluorescence (PF) and the corresponding decay curve for DNA-AgNCs embedded in poly(vinyl alcohol) (PVA) can be seen in the 20 to 40 ns time window in Fig. 1C. A second pulse, coming from the same continuum laser with a tunable NIR wavelength (690 nm to 1100 nm), is delayed about 46 ns with an optical fiber in order to transfer some of the DNA-AgNCs from the dark state into the S_1_ state, resulting in OADF. The OADF decay can be seen in Fig. 1C in the time range from 60 to 80 ns. However, we demonstrated previously that, even without primary visible excitation, a certain probability exists to pump DNA-AgNCs *via* a dark state-mediated consecutive photon absorption process into the S_1_ state, as shown in Fig. 1D.^10^ Comparing the secondary fluorescence amplitude (60 to 80 ns time range) shown in Fig. 1C and D, we can conclude that UCF is less efficient than OADF at 690 nm. To unravel the wavelength-dependent efficiencies of OADF and UCF, we measure the secondary fluorescence consisting of OADF and UCF signal at a constant primary excitation power while raster scanning the sample to avoid artifacts from potential photobleaching. Afterwards, we block the primary excitation light and measure the UCF signal by scanning the same region with only the secondary NIR excitation laser. UCF and OADF signals are both normalized by the excitation power of the secondary laser (0.5–2 kW cm^–2^). The resulting wavelength dependencies can be seen in Fig. 1E. All values in Fig. 1E are corrected by subtracting the constant offset in the time window, which mainly consists of detector dark counts and after pulsing. The OADF signal is separated from the UCF signal by subtracting the UCF signal from the secondary fluorescence. Fig. 1E shows that UCF requires higher photon energies than OADF as the UCF signal is barely observable at wavelengths above 800 nm. The OADF efficiency drops strongly between 850 and 900 nm and is rather constant from 1000 nm to 1100 nm. This offers the opportunity to deplete the dark state through OADF with minimal UCF, a basic requirement for potential background-free optical nanoscopy in a STED-like approach.^18^

**Fig. 2:**
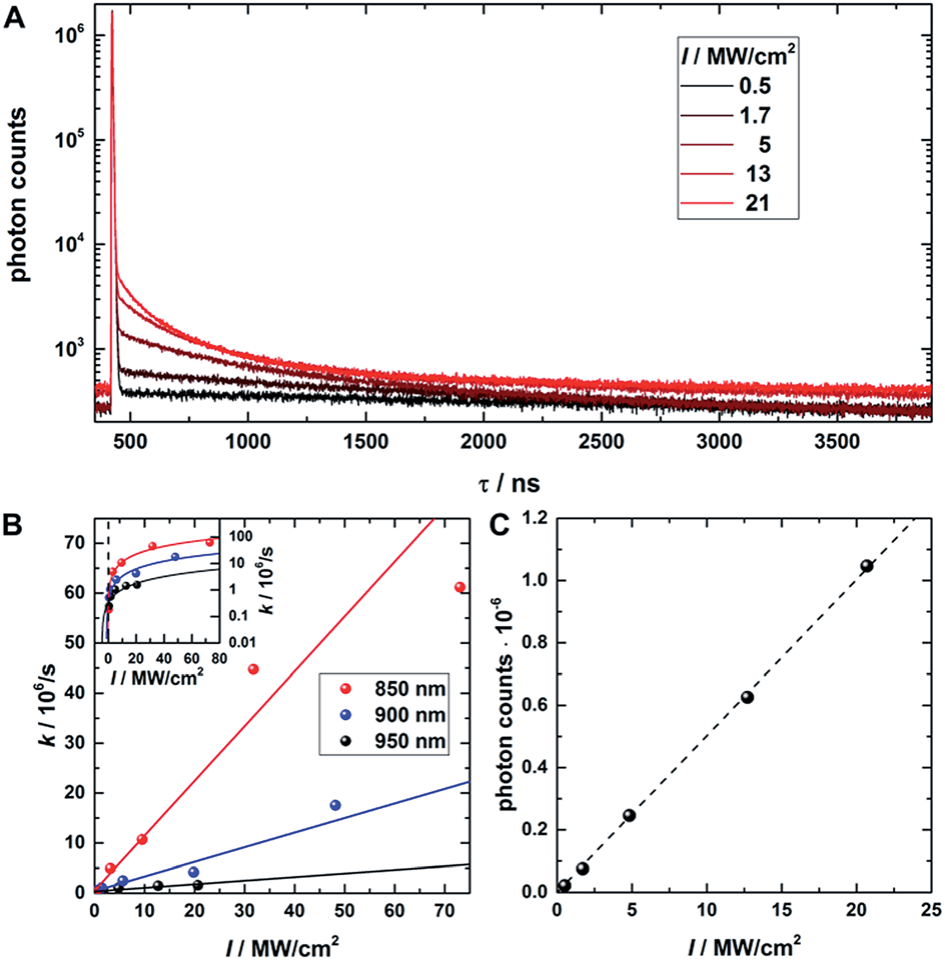
(A) Fluorescence decay curve of PVA-embedded DNA-AgNCs excited with a pulsed 560 nm laser and co-illuminated with CW 950 nm light. The initial fast, few nanoseconds decay component results from PF. The slow, hundred nanoseconds to microseconds decay components stem from depletion of the dark state by CW NIR illumination and the resulting OADF. This slow decay is here termed the depletion decay. (B) Average depletion decay rates extracted from tail-fitting the depletion decay component as a function of 850 nm, 900 nm and 950 nm laser intensities. The inset shows the same data on a logarithmic scale to highlight the *Y*-axis intercept. The data is fitted linearly (solid lines). (C) UCF intensity as a function of 950 nm CW laser intensity (without primary excitation). The data is fitted linearly (dashed line). Time-correlated single photon counting histograms of the actual data can be seen in Fig. S2.†

In a next step, a powerful 950 nm continuous wave (CW) Ti:sapphire laser was used as secondary NIR excitation source. Fig. 2A shows the PF decay upon excitation with a 560 nm pulse (250 kHz repetition rate) at 430 ns. In parallel, the sample was continuously illuminated with CW 950 nm light at different excitation intensities. The additional CW intensity results in very long additional multi-exponential decay components (which we will refer to as the average depletion decay time) with small amplitudes. A shortening of the average depletion decay time can be observed when increasing the CW intensity from 0.5 MW cm^–2^ to 21 MW cm^–2^. We have extracted the average depletion decay times by mono-, bi- or tri-exponential tail-fitting the part of the decay curve after the PF decay (see ESI for details†), followed by calculating the intensity-averaged depletion rate (see Fig. 2B). Extrapolating the average depletion rate to zero secondary (CW) intensity yields the inverse of the average dark state decay time 1/). Extrapolating the average depletion rate to zero secondary (CW) intensity yields the inverse of the average dark state decay time 1/〈*τ*〉_nr(D)_ (see ESI†), probed under these experimental conditions. This procedure is similar to the approach reported previously by Fleischer *et al.*^9^ However, in contrast to Fleischer *et al.*,^9^ we used much higher depletion intensities (about three orders of magnitude) and have opted to present the intensity averaged depletion decay time. Fitting the depletion decays in Fig. 2A multi-exponentially did not result in linear power dependencies for all individual decay components. Therefore, we used the intensity averaged depletion decay rate in this case. The use of an average dark state decay rate, which implies a range of dark state decay times, is supported by the UCF spectra showing spectral selection upon red-shifted excitation wavelengths (see below). These spectra indicate that a whole range of conformational states with potentially different dark state energy levels and dark state decay times might be frozen out in the polymer film. An additional argument is that the dark states, that we are probing in the 0.5 MW cm^–2^ to 21 MW cm^–2^ depletion regime, are most likely not the ones that are very efficiently undergoing OADF. This can be seen later in Fig. 4A. Using a NIR read-out pulse, about 40 percent of the dark states were already depleted by OADF after 46 ns at 0.7 MW cm^–2^. Heterogeneities in the OADF efficiency were also shown to be present at the single molecule level, most likely again due to freezing out of specific conformations in the PVA polymer film.^11^ Besides using a wavelength of 950 nm, the same experiment was also performed using 900 and 850 nm as secondary NIR excitation wavelengths. All three applied secondary wavelengths result in similar Besides using a wavelength of 950 nm, the same experiment was also performed using 900 and 850 nm as secondary NIR excitation wavelengths. All three applied secondary wavelengths result in similar 〈*τ*〉_nr(D)_ values in the range of 2 to 2.5 μs, which are in good agreement with previously reported dark state decay times of other DNA-AgNCs.^9^,^14^,^16^,^17^ Future experiments performed at every wavelength and over a larger depletion intensity range and/or time-gated detection of the OADF spectra could provide more precise information on the number and/or distribution of decay times of the dark state. Even though the UCF signal is close to zero in Fig. 1E, we can observe UCF at 850, 900 and 950 nm for higher intensities in the range of tens of MW cm^–2^. This can be seen from the offset rise of the decay curves with increasing CW excitation intensities, as displayed in Fig. 2A. To further analyze the increase in UCF, we measured UCF intensities without primary excitation (see Fig. S2†), which are plotted in Fig. 2C as a function of CW excitation intensity for the 950 nm experiment. The data shows clearly a linear trend in the experimental excitation intensity regime, which supports the assumption of a consecutive photon absorption process (see ESI for more details†) *via* the dark state, and rules out the possibility of a coherent two-photon (quadratic) absorption process.^10^,^20^,^21^ We like to point out that “hot band” excitation of thermally (Boltzmann) populated states also gives rise to anti-Stokes emission and a linear power dependence, but we consider this process to be unlikely due to the large energy difference between the absorption maximum of the DNA-AgNC and the wavelengths of the secondary laser used in the experiments.^22^

### Spectral relaxation beyond the PF decay time

**Fig. 3:**
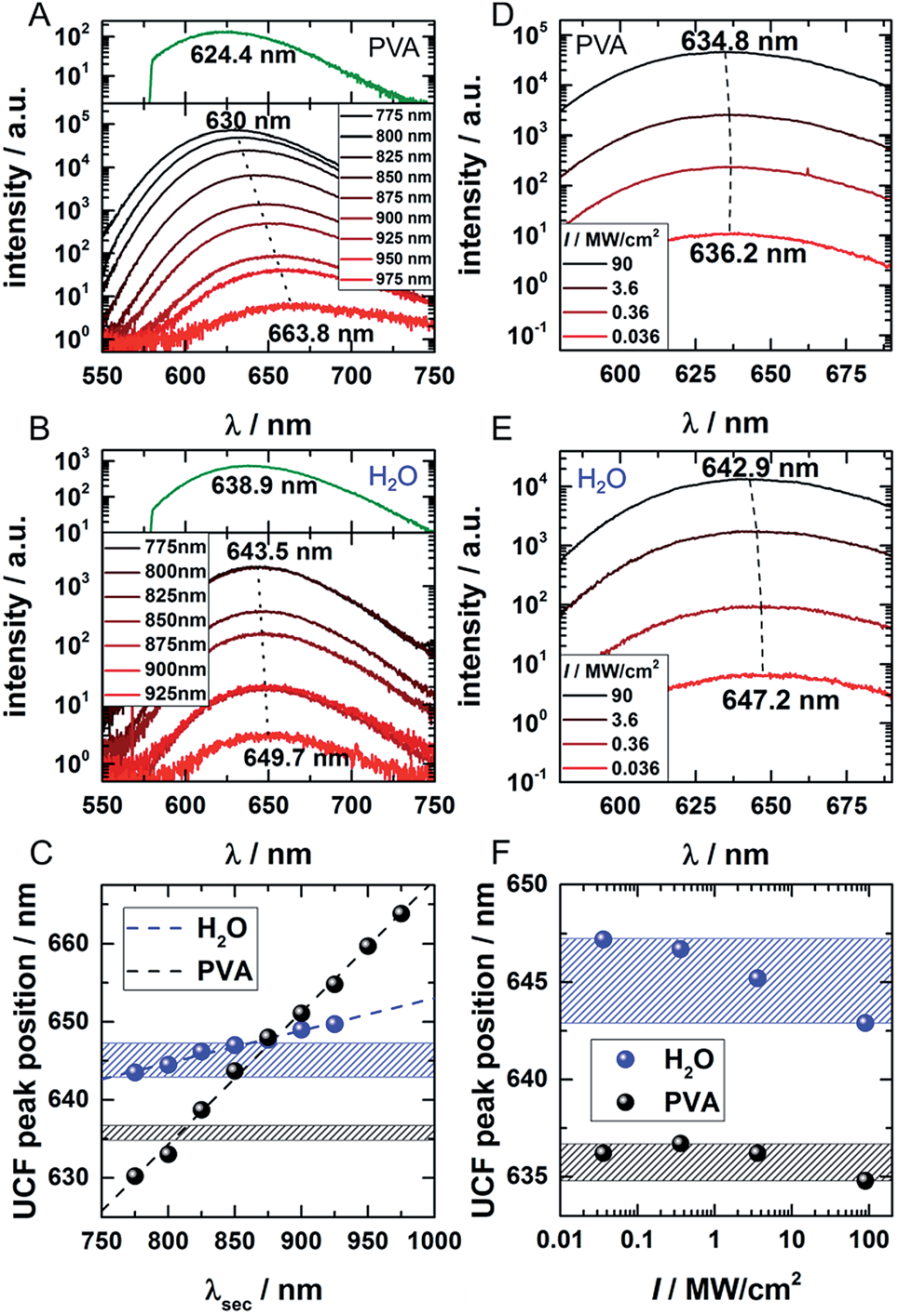
(A and B) UCF spectra for DNA-AgNCs embedded in PVA and in water, respectively, for different NIR wavelengths (CW excitation 775–975 nm, 16 mW for 775–950 nm, 6 mW for 975 nm). The dotted lines connect the UCF maxima of the spectra resulting from the shortest and longest applied wavelengths. The PF upon 560 nm excitation (dark green spectra) is given in the top part of the graphs for comparison to the UCF spectra. (C) UCF peak position for DNA-AgNCs in PVA (black) and water (blue) as a function of the applied NIR wavelength. (D and E) UCF spectra of DNA-AgNCs in PVA and water, respectively, for different excitation intensities of 800 nm excitation light. The dotted lines connect the peak positions of the spectrum acquired at lowest and highest excitation intensities, respectively. (F) UCF peak position for DNA-AgNCs in PVA (black) and water (blue) as a function of 800 nm laser intensity. The ranges of UCF peak positions obtained in water (blue marked zone) and PVA (black marked zone) are also drawn in (C) for comparison.

In order to investigate the UCF signal in more detail, we acquired UCF emission spectra for different NIR excitation wavelengths and intensities. Surprisingly, we observed a clear and gradual red-shift of the UCF spectrum for increasing NIR excitation wavelengths in comparison to the PF emission spectrum (excitation at 560 nm). This UCF spectral shift is particularly strong for DNA-AgNCs embedded in PVA and less pronounced in solution (Fig. 3A–C). In PVA, the cause of the red-shift with longer NIR excitation wavelength is mainly due to freezing of a large range of spectral (conformational) states of the DNA-AgNCs during the immobilization process in PVA – a process which is well known for organic dyes.^23^,^24^ The findings in PVA can be explained with the wavelength dependence of UCF shown in Fig. 1E. Since UCF drops almost exponentially with wavelength from 800 nm onwards, it is likely that UCF will be selective for more and more red-shifted frozen spectral conformations upon increasing the NIR excitation wavelength.

In this case, we make the assumption that dark state, emissive state and ground state cannot spectrally relax significantly (by conformational rearrangement of the structure). Therefore, it is very likely that long wavelengths preferably select frozen conformations with a low energy of the dark state which then leads to a lower energy gap between the emissive and ground state (several scenarios are possible to achieve this). In solution the spectral relaxation should be free (no conformational freezing) and hence only limited by the time the molecule spends in the dark and excited states.^25^–^27^ To test our hypothesis and demonstrate the difference between the solution and PVA case, UCF spectra were recorded for varying excitation intensities at a fixed wavelength of 800 nm (Fig. 3D and E). For the solution case, and at low excitation intensities, the DNA-AgNCs in the dark state have more time to relax before they are transferred to the emissive state. It seems that the inefficient UCF at low excitation intensities allows the DNA-AgNCs to spectrally relax longer in the dark state and yield a more red-shifted UCF spectrum. Increasing the secondary excitation intensity shortens the time spent in the dark state and blue-shifts the UCF spectra close to what is observed for the normal PF emission spectrum (see Fig. 3B and E). This argument is plausible because the range of UCF peak positions (blue areas in Fig. 3C and F), which can be obtained by either changing the NIR excitation wavelength or the NIR excitation intensity, is similar in water. Since spectral relaxation is strongly hindered in a rigid polymer matrix such as PVA, one would not expect to see such a change upon increasing the secondary laser intensity. This is indeed the case and only a negligible shift is present, as shown in Fig. 3F (black area) in comparison to the secondary laser wavelength-dependent shift (black dots) shown in Fig. 3C. It is important to reiterate that the spectral relaxation in solution (on the nanoseconds and slower time scale) results from conformational changes of the DNA scaffold upon charge redistribution in the dark and emissive state.^25^–^27^ The fast electronic spectral relaxation, accounting for the majority of the Stokes shift, happens on a sub-picosecond timescale and is not being discussed here.^13^,^15^,^19^,^20^ The slow, nanosecond spectral relaxation of the emissive state, which occurs in solution, has been shown previously by time-correlated single photon measurements and can be modulated by changing the viscosity of the medium or the temperature.^27^,^28^ Intriguingly, UCF allows us to probe this slow spectral relaxation beyond the normal PF decay time, by pre-relaxing the excited state up to several microseconds into the dark state. This interesting trick could help to further understand the structural and conformational origins of this process in the near future.

### Dark-state depletion towards OADF nanoscopy

**Fig. 4:**
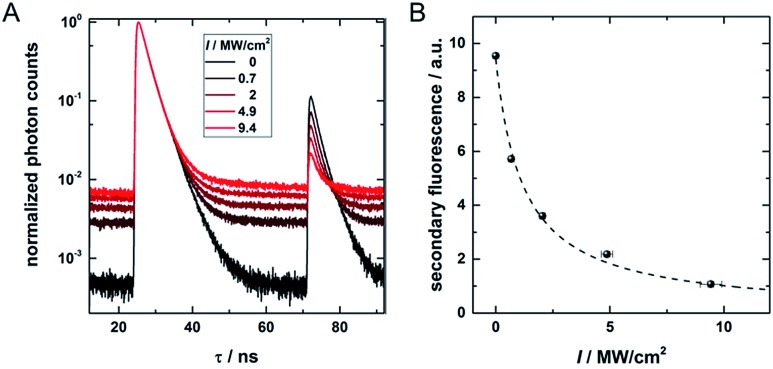
(A) Decay curves containing pulsed primary emission (PF, 24–50 ns), pulsed secondary emission (OADF + UCF, 71–85 ns) and CW OADF + UCF at different depletion intensities from a continuous wave 960 nm depletion laser. (B) Secondary fluorescence (71.3–74.5 ns region) as a function of the CW 960 nm laser intensity. The offset, determined in the time window from 60 to 64 ns, is subtracted.

To check the potential of CW dark-state depletion for STED-like OADF nanoscopy, we used a setup similar to the one presented in Fig. S1.† It contains a CW 960 nm depletion laser, a pulsed 560 nm primary laser (0.73 kW cm^–2^) and a pulsed 765–860 nm delayed secondary read-out laser (17.5 kW cm^–2^), both with a repetition rate of 11.13 MHz. In this case, the latter two were not provided from an acousto-optic tunable filter but from the direct fiber output of the continuum laser source. The wavelength selection was achieved through band pass filters. The Gaussian output beams of all three wavelengths were overlapped in the focal plane. The role of the CW 960 nm laser is to continuously depopulate the dark state. If the CW beam-profile is changed from Gaussian- to doughnut-shape, the setup is similar to a previously described STED-like setup that should yield sub-diffraction spatial resolution.^18^,^29^ Fig. 4A shows decay curves for PF and OADF at different 960 nm CW excitation intensities ranging from 0 to 9.4 MW cm^–2^. Two main findings emerge from the data: (1) as expected, the amplitude of the OADF from the secondary read-out pulse is reduced when increasing the 960 nm CW intensity (see Fig. 4B), and (2) the offset increases with CW laser intensity (see Fig. 4A) due to UCF. This increase of UCF signal currently limits us from using the OADF from the secondary read-out pulse to yield sub-diffraction resolution images. Further research on other DNA-AgNCs might yield different OADF-to-UCF efficiencies and might enable STED-like OADF-based nanoscopy.

## Experimental

### Optical setup

The experimental setup is schematically depicted in Fig. S1 in the ESI.† Time-correlated single photon counting data and spectra were acquired with a home-built confocal microscope.^30^ For the NIR excitation spectroscopy, the visible, primary (560 nm), and NIR, secondary (690–1100 nm), excitation lines were selected from a pulsed white light laser (NKT SuperK EXTREME EXB-6) producing continuous emission from 420 to 2400 nm by means of an acousto-optic tunable filter (NKT SuperK SELECT AOTF). Since the NIR output of the white light laser is strongly wavelength-dependent, the secondary excitation intensity could only be kept at values in a range from 0.5 to 2 kW cm^–2^. The intensity was determined with a power meter (Thorlabs) and used for normalization of the OADF/UCF signal. The NIR secondary excitation line was coupled into a 10 m polarization maintaining fiber (Thorlabs, P1-780PM-FC-10), which delayed the NIR pulse by about 46 ns with respect to the visible primary excitation pulse. The visible primary excitation beam was cleaned up by a 561 nm band pass filter (Semrock MaxLine Laser Line) and a 633 nm short-pass (Semrock), while the secondary pulsed NIR probe beam was cleaned with a 647 nm long pass filter (Semrock). Both beams were coaligned by transmission/reflection through/from a 650 nm dichroic mirror DMLP650R, Thorlabs.

The optional depletion beam with a tunable wavelength from 775 to 975 nm was provided by a continuous wave Ti:sapphire laser (Spectra Physics 3900S). The laser light was sent through a 10 m polarization maintaining single mode fiber (Thorlabs, P1-780PM-FC-10) for cleaning up the beam profile. A combination of a 647 nm long pass filter (Semrock) and, depending on the actual wavelength, a 937 nm, 815 nm or 785 nm long pass filter removed residual side bands, while a Glan–Thompson polarizer (Thorlabs) with an extinction ratio of 100 000 : 1 was used to obtain linearly polarized light. Afterwards, the beam was extended by a telecentric lens system. The polarization of the resulting depletion beam was set to circular in the focal plane by a combination of *λ*/2 and *λ*/4 wave plates (Thorlabs). Primary and secondary pulsed beams, as well as the CW depletion beam, were recombined by a 50 : 50 non-polarizing beam splitter cube (Thorlabs) and then reflected by a 30 : 70 beam splitter (XF122 Omega Optical) into an oil-immersion objective (Olympus, UPlanSApo 100×, NA = 1.4). The objective focused the beams onto the sample and collected the fluorescence signal. The sample was scanned with a piezo scanner (Physik Instrumente). Primary and secondary laser light was blocked by a 561 nm long-pass filter (Semrock Edge Basic) and a 700 nm short-pass filter (Chroma, ET700SP-2P8) in the detection path for the experiments in Fig. 1, 2 and 4. For recording the UCF spectra in Fig. 3, a 700 nm or 750 nm short-pass filter was used. The fluorescence signal was detected by an avalanche photodiode (PerkinElmer CD3226) being connected to a single photon counting module (Becker & Hickl SPC-830). Data were analyzed with self-written Matlab algorithms. For the pulsed measurements, all stated laser intensities are average intensities, not peak intensities.

### DNA-AgNC synthesis and purification

DNA-AgNCs were synthesized and purified as described by Cerretani *et al.*^27^ The DNA-AgNCs have an absorption and emission maximum of 573 nm and 640 nm, respectively, in a 10 mM NH_4_OAc solution at 25 °C. At this temperature, the fluorescence quantum yield is 0.80 and the intensity-weighted average decay time is 2.59 ns.^27^ The stock solution of DNA-AgNCs was diluted in a solution of PVA (Sigma Aldrich) and Milli-Q water until it reached a concentration of about 10^–8^ M. The solution was drop-cast onto an annealed glass coverslip (Menzel). For measuring DNA-AgNCs in water, a ∼10^–7^ M dilution of the stock solution was prepared with nuclease free water (IDT). A 50 μl droplet was then deposited onto a clean cover slide.

## Conclusions

We have used NIR excitation spectroscopy as a tool to disentangle optically activated delayed fluorescence (OADF) and upconversion fluorescence (UCF) in DNA-AgNCs. Thereby, we found that UCF can be most efficiently generated at wavelengths below 800 nm, while OADF can still be observed at moderate intensities up to 1100 nm – the longest wavelength available in this study.

However, the intensity values required to fully deplete the dark state population through OADF lead again to UCF which increases linearly with excitation intensity – a clear sign of consecutive photon absorption. Investigation of the average depletion rates and extrapolation to zero secondary laser intensity yielded average dark state lifetimes in the range of a few microseconds, in agreement with previous results on other DNA-AgNCs.^9^,^14^,^16^,^17^

The observed red-shift of the UCF spectra with longer secondary excitation wavelengths in a rigid polymer matrix can be explained by spectral selection of frozen conformations of DNA-AgNCs. From the measured red-shift of clusters in water at reduced secondary excitation power, we conclude that slow spectral relaxation, due to conformational reorientation, takes also place while the DNA-AgNCs are in the dark state.

Finally, we have shown the feasibility of dark state depletion under continuous wave irradiation. This gives the opportunity for future super resolution applications of OADF with DNA-AgNCs.

## Conflicts of interest

There are no conflicts to declare.

## Supplementary Material

Supplementary information
