# Supplementary material for: Disentangling optically activated delayed fluorescence and upconversion fluorescence in DNA stabilized silver nanoclusters
Source: Chem Sci. 2019 Apr 24;10(20):5326–31. doi: 10.1039/c9sc00865a (PMC6540914; doi:10.1039/c9sc00865a)
Supplement: Supplementary file 1 [file SC-010-C9SC00865A-s001.pdf]

Electronic Supplementary Material (ESI) for Chemical Science.
This journal is © The Royal Society of Chemistry 2019

Supporting Information

Disentangling Optically Activated Delayed Fluorescence and Upconversion Fluorescence in DNA Stabilized Silver Nanoclusters.

Stefan Krause*, Cecilia Cerretani and Tom Vosch*

Nanoscience Center and Department of Chemistry, University of Copenhagen, Universitetsparken 5, 2100 Copenhagen, Denmark. Email: stefan.krause@chem.ku.dk, tom@chem.ku.dk

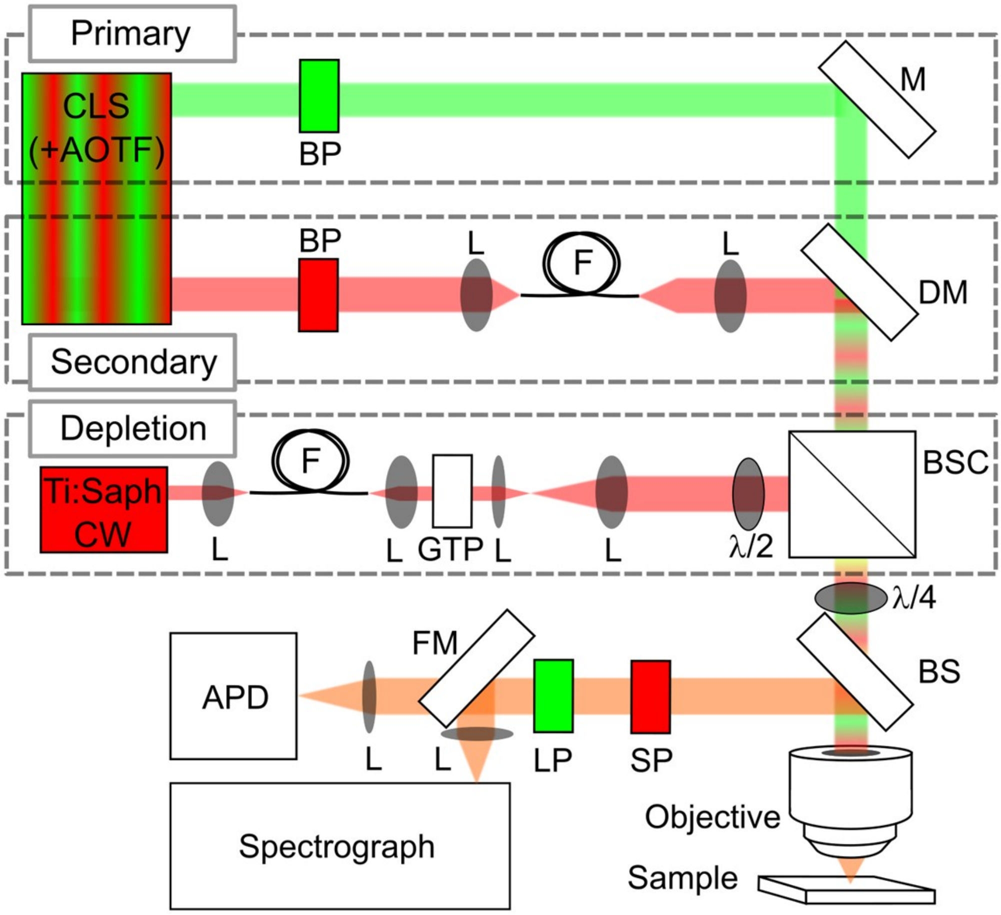

Figure S1: Scheme of the experimental setup consisting of a continuum laser source (CLS) with optional acousto-optic tunable filter (AOTF) for primary and secondary excitation. The visible (green) and NIR (red) part provide the primary excitation pulse at 560 nm and the secondary read-out pulse that can be tuned from 690 to 1100 nm. The secondary laser is delayed by 46 ns by means of an optical fiber (F). The depletion path consists of a continuous wave Ti:Sapphire laser, lenses (L), a fiber (F), a Glan-Thompson polarizer (GTP) and quarter/half wave plates. Primary, read-out and depletion beams are combined with a beam splitter cube (BSC). The fluorescence light is collected by an objective, cleaned by short (SP) and long pass (LP) filters and detected by an avalanche photo diode (APD), or a spectrograph via a flip mirror (FM), when recording PF or UCF spectra.

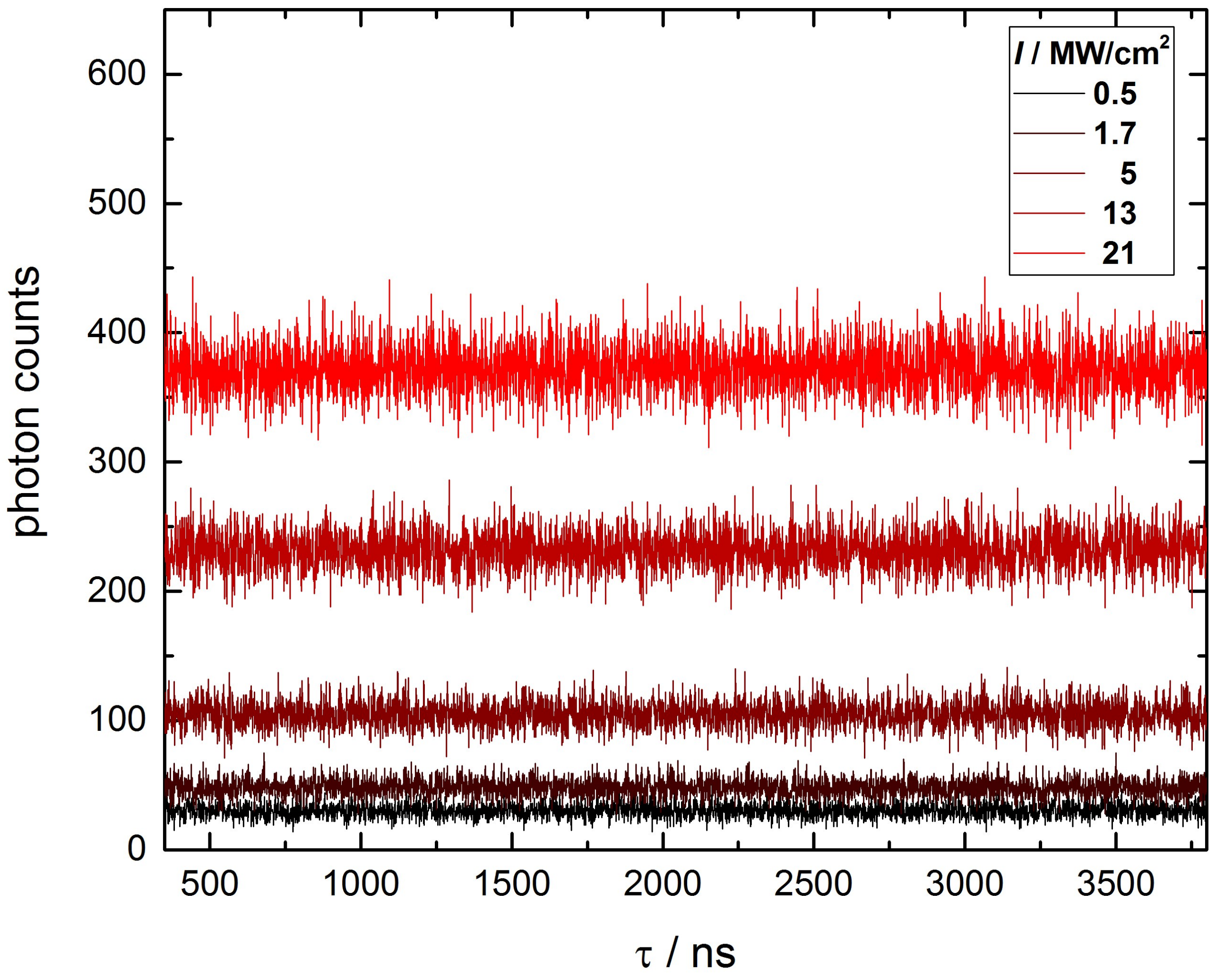

Figure S2: Single photon counting histograms, visualizing the UCF intensity without primary excitation for different CW excitation intensities at 950 nm.

Multi-exponential decay fitting results for <*k*>$_{DEPL}$ in Figure 2.

Due to the fact that the decay curves can be fitted satisfactory using mono- to tri-exponential fits, we have opted to present the data by the average depletion decay time instead of using the individual components. The tail-fitting started for all experiments at channel 443 (454.248 ns in the TAC window).

950 nm

I / MW/cm^2	A_1	t_1 / ns	A_2	t_2 / ns	A_3	t_3 / ns
0.5	1.8E-4	4016	-	-	-	-
1.7	8.5E-6	106	2.1E-4	1777	-	-
5	3.9E-5	188	2.6E-4	796	8.9E-4	53510
13	4.2E-4	73	8.6E-4	225	4.3E-4	959
21	0.0013	65	0.0013	229	2.65E-4	1194

900 nm

I / MW/cm^2	A_1	t_1 / ns	A_2	t_2 / ns	A_3	t_3 / ns
0.6	1.6E-4	384	2.4E-4	2127	-	-
1.4	5.3E-4	232	3.6E-4	1164	-	-
5.7	0.002	104	7.0E-4	571	-	-
20	0.005	65	0.001	385	-	-
48	0.013	19	0.007	75	-	-

850 nm

I / MW/cm^2	A_1	t_1 / ns	A_2	t_2 / ns	A_3	t_3 / ns
0.3	1.5E-8	122	5.7E-4	5501	-	-
3.1	8.3E-4	55	0.0011	230	-	-
9.5	0.005	30	0.0028	122	-	-
32	2.9E-4	2.1	0.0064	22	-	-
73	0.0018	4	0.012	17	-	-

Determination of the average dark state decay rate (<k>$_{nr(D)}$).

The CW laser in Figure 2 will continuously deplete the dark state population and generate OADF. This depletion of the dark state will appear in the time window as multiple, CW excitation intensity-dependent decay components (tens of nanoseconds to tens of microseconds). The origin of the multiple decay components, needed to satisfactorily tail-fit the decay curve, could be due to many reasons. These include multiple separate electronic dark states,[1] a single electronic dark state that has a broad distribution of photophysical

parameters, depending on different DNA and AgNC conformations, and/or a broad distribution of different OADF efficiencies.[1,2] We would also like to point out that the dark states probed in this way might be dependent on excitation intensity (once the depletion rate reaches the PF rate, it will be swamped by the PF signal and only less efficiently depleted dark states will be visible as a long tail) and wavelength (in case different dark states have different wavelength-dependent efficiencies). For the determination of the intensity-averaged dark state decay rate probed under the experimental conditions,

$$< k >_{nr(D)} = \sum_i \frac{A_i t_i^2}{A_i t_i}$$

we are interested in the extrapolation towards zero CW excitation intensity. Hence, we can write the intensity-averaged depletion rate ($<k>_{DEPL}$) as:

$<k>_{DEPL} = <k>_{nr(D)} + <k>_{OADF}$

$<k>_{OADF}$ is dependent on the CW laser intensity (I_{sec}) and can be generally written as:

$<k>_{OADF} = (I_{sec} \cdot <\sigma>_{OADF} \cdot \lambda)/h \cdot c$

with $<\sigma>_{OADF}$ = average cross-section for OADF, λ = wavelength of the light, h = Planck's constant and c = speed of light.

Substitution in the first equation gives:

$<k>_{DEPL} = <k>_{nr(D)} + (I_{sec} \cdot <\sigma>_{OADF} \cdot \lambda)/h \cdot c$

Showing that $<k>_{DEPL} = <k>_{nr(D)}$ when $I_{sec} = 0$

Excitation intensity dependence of the UCF.

In the following section we will explain why UCF, when it is clearly observed (Figure S2 and Figure 2C), can be approximated to be linear with excitation intensity.

Based on the scheme in Figure 1B, we can redraw the relevant states and transitions involved in the UCF process. The calculations assume a steady-state experiment with continuous wave NIR illumination.

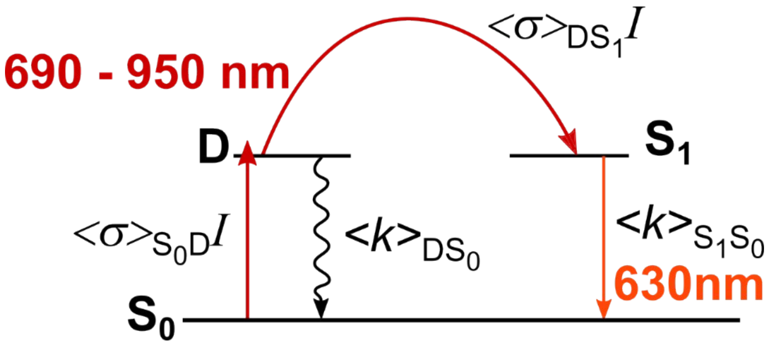

For the steady-state CW case in Figure S2, we can set up the following equations with $\langle\sigma\rangle$ the average cross section, $\langle k\rangle$ the average rate constant, *I* the laser intensity, λ = wavelength of the light, *h* = Planck's constant and *c* = speed of light, $<k>_{nr(D)}$ is renamed to $\langle k\rangle_{DS_0}$ and $k_{nr}(S1)+ k_r(S1)$ is renamed to $\langle k\rangle_{S_1S_0}$.

$$0 = \frac{d[D]}{dt} = [S_0]\langle\sigma\rangle_{S_0D} I\frac{\lambda}{hc} - \langle k\rangle_{DS_0}[D] - [D]\langle\sigma\rangle_{DS_1} I\frac{\lambda}{hc}$$

$$0 = \frac{d[S_1]}{dt} = [D]\langle\sigma\rangle_{DS_1} I\frac{\lambda}{hc} - \langle k\rangle_{S_1S_0}[S_1]$$

Hence:

$$[D] = \frac{[S_0]\langle\sigma\rangle_{S_0D} I\frac{\lambda}{hc}}{\left(\langle k\rangle_{DS_0} + \langle\sigma\rangle_{DS_1} I\frac{\lambda}{hc}\right)}$$

$$[S_1] = \frac{[S_0]\langle\sigma\rangle_{S_0D} I\frac{\lambda}{hc}\langle\sigma\rangle_{DS_1} I\frac{\lambda}{hc}}{\langle k\rangle_{S_1S_0}\left(\langle k\rangle_{DS_0} + \langle\sigma\rangle_{DS_1} I\frac{\lambda}{hc}\right)}$$

When $\langle\sigma\rangle_{DS_1} I\frac{\lambda}{hc} >> \langle k\rangle_{DS_0}$ the equation simplifies to a linear dependency:

$$[S_1] = \frac{[S_0]\langle \sigma \rangle_{S_0 D} I \frac{\lambda}{hc}}{\langle k \rangle_{S_1 S_0}}$$

Practically, if $\langle \sigma \rangle_{DS_1} I \frac{\lambda}{hc} < \langle k \rangle_{DS_0}$ the dark state decay would outcompete the UCF process and it would be hard to detect UCF. Figure 4 shows that even for the lowest depletion intensity used (0.7 MW/cm^2), after 46 ns, about 40 percent of the dark states formed by the primary pulse are depleted. Here, we make the assumption that the OADF process is the same process as the second step in the UCF process. If this assumption holds, the intensity range of the CW laser that we are using is capable of depleting the dark states efficiently and hence we can assume that ($\langle \sigma \rangle_{DS_1} I \frac{\lambda}{hc} >> \langle k \rangle_{DS_0}$). As a result of this, we can assume that we are in the linear regime and fit our UCF with a linear function (although, as mentioned, this is an approximation of the equation).

[1] B. C. Fleischer, J. T. Petty, J.-C. Hsiang and R. M. Dickson, J. Phys. Chem. Lett., 2017.

[2] S. Krause, M. R. Carro-Temboury, C. Cerretani and T. Vosch, Physical Chemistry Chemical Physics, 2018, 20, 16316-16319.
